# Is there an association with phosphorylation and dephosphorylation of Src kinase at tyrosine 530 and breast cancer patient disease-specific survival

**DOI:** 10.1038/sj.bjc.6605913

**Published:** 2010-11-09

**Authors:** B Elsberger, B A Tan, E A Mallon, V G Brunton, J Edwards

**Affiliations:** 1 Institute of Cancer, College of Medical, Veterinary and Life Sciences, University of Glasgow, McGregor Building, Western Infirmary, Glasgow G11 6NT, UK; 2 Department of Pathology, Glasgow Western Infirmary, Dumbarton Road, Glasgow G11 6NT, UK; 3 Edinburgh Cancer Research Centre, University of Edinburgh, Crewe Road, Edinburgh EH4 2XR, UK

**Keywords:** Src kinase, breast cancer, immunohistochemistry, disease-specific survival

## Abstract

**Background:**

Recent work has demonstrated that c-Src and fully activated Y419Src expression was associated with poor clinical outcome of breast cancer patients. It is unknown whether different activation stages of c-Src equally influence disease-specific survival of breast cancer patients.

**Methods:**

Immunohistochemistry was performed on 165 resected breast cancers using antibodies to phosphorylated and dephosphorylated Src kinase tyrosine site 530. Expression was assessed using the weighted histoscore method.

**Results:**

Majority of phosphorylated and dephosphorylated Y530Src expression was observed in the nucleus and cytoplasm. Only 3.6% of phosphorylated Y530Src (pY530Src) expression was detected in the membrane, compared with 53% with dephosphorylated Y530Src. Nuclear expression of pY530Src correlated negatively with oestrogen receptor (ER) status (*χ*^2^ *P*<0.001), whereas cytoplasmic phosphorylated and dephosphorylated Y530Src expression correlated negatively with membrane c-Src expression (*χ*^2^ *P*=0.008, *χ*^2^ *P*<0.001). On univariate and multivariate analysis, no significant association was noticed between phosphorylated or dephosphorylated Y530Src expression and disease-specific survival at any cellular location.

**Conclusion:**

ER-negative breast cancer patients were more likely to express pY530Src in the nucleus. Breast cancer patients with higher cytoplasmic expression of phosphorylated or dephosphorylated Y530Src were more likely not to express c-Src at the membrane. Phosphorylated and dephosphorylated Y530Src expression is not associated with survival of patients.

Src kinase is implicated as a regulator of cell proliferation and survival, and as a contributor to cellular migration and invasion (Frame, 2002). Recent work has demonstrated that cytoplasmic c-Src and fully activated membrane Src phosphotyrosine 419 expression in breast cancer specimens was associated with poor clinical outcome (Elsberger *et al*, 2009). c-Src is activated by a number of pathways. The classical activation pathway includes dephosphorylation of Y530 to initiate a configuration change of the protein (partial activation), allowing full activation by autophosphorylation of tyrosine site 419. Phosphorylation of the tyrosine residue 530 on the c-terminal tail by Csk tyrosine kinase acts as a negative regulatory protein-binding site, keeping Src kinase in a closed confirmation (Roskoski Jr, 2005) and inactive. The Src kinase can be found at different subcellular locations. It is most abundantly localised in the cell cytoplasm, but is re-localised to the membrane when activated. Subcellular localisation has also been suggested to regulate Src activity (Bjorge *et al*, 2000). In studies examining the role of Src in assembly of focal adhesions, the inactive form of Src was localised to the perinuclear region of cells in association with microtubules. On activation, Src was transported to the plasma membrane where it is recruited to focal adhesions (Fincham *et al*, 2000).

The aim of this translational study was to assess associations between different stages of Src activation and expression pattern with clinicopathological features of the cohort, cellular distribution and their influence on breast cancer patients' disease-specific survival.

## Materials and methods

### Patients

A total of 314 patients were initially recruited. All patients were diagnosed with operable invasive breast carcinoma between 1980 and 1999 in the Greater Glasgow area. These patients received standard adjuvant treatment according to protocols at the time of diagnosis. We included patients in our analysis only when all clinical data, previous published c-Src expression data (Elsberger *et al*, 2009) and phosphorylated and dephosphorylated Y530Src kinase expression data were available (*n*=165). Ethics approval was granted by the local ethics committee.

### Tissue microarray construction

Tissue microarrays (TMAs) were already available for use in this study. Cores of breast cancer tissue (0.6 mm^2^), identified by the pathologist, were removed from representative areas of the tumour taken from breast cancer patients at time of surgical resection. All TMA blocks were constructed in triplicate.

### Immunohistochemistry

Staining for ER (oestrogen receptor), PR (progesterone receptor) and HER2 (human epidermal growth factor receptor) had been previously performed for the cohort (Tovey *et al*, 2005). To investigate the different stages of Src kinase activation, Clone28 antibody (Invitrogen, Paisley, UK) was employed to recognise the partially activated form of Src, as Clone28 recognised Src that is unphosphorylated at Y530, independent of the phosphorylation status of Y419. To evaluate protein expression of inactive Src kinase, anti-Src family negative regulatory [pY] site antibody (Invitrogen) was used for detecting phosphorylated tyrosine site 530 (pY530Src). Antigen retrieval was performed in a preheated antigen retrieval solution (citrate buffer (pH 6.0), Vector Laboratories, Burlingame, CA, USA; Peterborough, UK), followed by heating tissue sections under pressure for 5 min in a microwave. Endogenous peroxidase activity was blocked by incubation in 3% hydrogen peroxide. To reduce nonspecific binding, the tissue sections were then incubated with 5% horse serum (Vector Laboratories) in antibody diluent (DAKO Cytomation, Glostrup, Denmark) for 20 min at room temperature. Slides were incubated with Clone28 (1 : 500) and anti-Src family negative regulatory [pY] site antibody (1 : 1500) overnight at 4°C. Signal was amplified and visualised using the DAKO Envision Kit (DAKO Cytomation) and DAB (Vector Laboratories). In each run, a positive and negative isotype-matched control was included to ensure no false-positive staining or intense stromal staining. Specificity of antibodies was confirmed by western blotting. Protein expression of each core was assessed using the weighted histoscore method (Kirkegaard *et al*, 2006). Over time and extensive section cutting of the TMAs, tissue/cores vanished, resulting in a decreased number for expression analysis. Each cellular location was independently assessed for phosphorylated and dephosphorylated Y530Src kinase expression. Agreement between observers was excellent and measured in interclass correlation coefficient (all ICCC >0.83).

### Statistical analysis

Medians of nuclear, cytoplasmic and membrane phosphorylated and dephosphorylated Y530Src expression were used for statistical analysis. Patients were split into groups with expression (above median) and no expression (below or equal to the median). Disease-specific survival rates were generated using the Kaplan–Meier method. The log-rank test was used to compare significant differences between subgroups using univariate analysis. To remove a variable from the model, the corresponding *P*-value had to be >0.05.

Inter-relationships among clinical parameters, phosphorylated and dephosphorylated Y530Src and c-Src expression were calculated using the *χ*^2^-test. The statistical analyses were performed using a statistical software package (SPSS 15.0 Inc., Chicago, IL, USA).

## Results

### Clinicopathological details

Clinicopathological features of the cohort are displayed in Table 1. Mean patient follow-up was 7.1 years (minimum follow-up was 2.1 years and the maximum follow-up was 20 years).

### pY530Src expression levels

**Figure 1:**
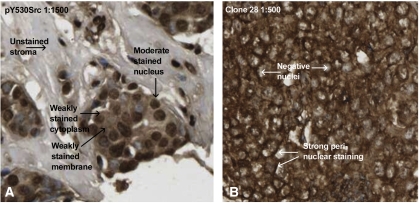
IHC pictures using anti-Src family negative regulatory [pY] site antibody and Clone28 antibody; magnification × 400. (**A**) Illustrates invasive breast cancer immunohistochemically stained with anti-Src family negative regulatory (pY) site antibody (Invitrogen, 1 : 1500); moderate staining of nuclei, weak staining of cell cytoplasm and membrane can be seen. (**B**) Demonstrates strong perinuclear staining in invasive breast cancer stained with Clone28 antibody (Invitrogen, 1 : 500).

Nuclear pY530Src expression was seen in the nucleus of 50% of tumours investigated, cytoplasmic expression was observed in 44% of tumours and only 3.6% of tumours investigated expressed pY530Src in the membrane (Figure 1A).

Only one significant correlation between clinicopathological features of the cohort and pY530Src expression was detected; ER status correlated negatively with nuclear expression of pY530Src (*χ*^2^ *P*<0.001). Interestingly, cytoplasmic pY530Src correlated negatively with membrane c-Src expression (*χ*^2^ *P*=0.008). No significant correlations were noted with membrane pY530Src expression. Inter-relationships between the different pY530Src cellular locations were insignificant. On univariate and multivariate analysis, there was no significant association noticed between pY530Src expression and disease-specific survival at any cellular location (nuclear *P*=0.192, cytoplasmic *P*=0.294 and membranous *P*=0.748).

### Dephosphorylated Y530Src kinase expression levels

Perinuclear staining of dephosphorylated Y530Src was prominent (Figure 1B) and was, therefore, included in the analysis. Nuclear dephosphorylated Y530Src expression was witnessed in the nucleus of 50% of tumours investigated and cytoplasmic expression was observed in 46% of tumours.

A remarkable 53% of tumours expressed dephosphorylated Y530Src in the membrane and 55% of tumours expressed perinuclear dephosphorylated Y530Src. No significant correlations were noted between clinicopathological features and any of the dephosphorylated Y530Src expression sites. Nuclear and membrane dephosphorylated Y530Src expression showed no significant correlations with any of the other c-Src and pY530Src expressions. Inter-relationship analysis of individual cellular locations demonstrated a positive correlation between nuclear and cytoplasmic dephosphorylated Y530Src expression (*χ*^2^ *P*<0.001), and a negative correlation between nuclear and perinuclear dephosphorylated Y530Src expression (*χ*^2^ *P*<0.001). As already observed with cytoplasmic pY530Src expression, cytoplasmic dephosphorylated Y530Src correlated negatively with membrane c-Src expression (*χ*^2^ *P*<0.001). Perinuclear dephosphorylated Y530Src expression correlated negatively with cytoplasmic pY530Src (*χ*^2^ *P*<0.001) and nuclear and cytoplasmic dephosphorylated Y530Src (both *χ*^2^ *P*<0.001). No significant association was noticed between dephosphorylated Y530Src expression and disease-specific survival at any cellular location (nuclear *P*=0.313, cytoplasmic *P*=0.810, membranous *P*=0.568 and perinuclear region *P*=0.625).

## Discussion

*In vitro* evidence for a role for c-Src in breast cancer is convincing, but hardly supported by translational clinical studies. A preceding study (Elsberger *et al*, 2009) revealed significant associations among cytoplasmic c-Src, phosphorylated activated Y419Src kinase expressed in the membrane with shorter disease-specific survival, increasing grade, tumour size, ER negativity and HER2 positivity. These results support the role of Src kinase expression and activation, currently described in the literature. To expand the assessment of various phosphorylation sites of Src, phosphorylated and dephosphorylated tyrosine site 530 was investigated in this study. These antibodies should represent c-Src in its inactive form and partially activated appearance. Clone28 has been used in previous studies (Sakai *et al*, 1998; Ito *et al*, 2001, 2002) to determine whether c-Src has a role in colorectal, hepatocellular or mammary tumorigenesis. Intense staining was frequently observed in colonic adenomas, as well as in well- and moderately differentiated hepatocellular carcinomas and in colonic adenocarcinomas at an early stage, suggesting that expression of Clone28 was linked to an early event in carcinogenesis *in situ* before invasive and metastatic elements manifest. Therefore, it is not surprising that none of the expression patterns of phosphorylated and dephosphorylated Y530 were significantly associated with disease-specific survival of mixed breast cancer patient cohort.

Phosphorylated and dephosphorylated Y530Src were more or less equally expressed in the nucleus and cytoplasm. However, there was a noteworthy divergence of their expression at the cell membrane. Phosphorylated Y530Src was rarely detected in the membrane compared with dephosphorylated Y530Src, which was mainly expressed in the membrane and perinuclear region, corresponding with features seen in Sakai's colonic tissue immunohistochemistry study (Sakai *et al*, 1998).

Interestingly, cytoplasmic phosphorylated (inactive form) and dephosphorylated Y530Src correlated negatively with membrane c-Src expression (site of activation), confirming validity of results regarding functional and cellular location, for example, the cell membrane being site of Src activation.

Another negative correlation was observed between nuclear expression of pY530Src and ER status (*χ*^2^ *P*<0.001), suggesting that ER-negative breast cancer patients are more likely to express inactive Src in the nucleus, valued as the site of inactivation. *In vitro* experiments with c-Src showed that the oestradiol receptor can activate c-Src, proposing that c-Src is an initial and integral part of the signalling events mediated by the oestradiol receptor (Migliaccio *et al*, 1996).

In this study, we determined that not only protein activation but also stage and site of activation along with cellular location have an important role for breast cancer patients. It also supports the hypothesis that dephosphorylated Y530Src is more related to early stages of carcinogenesis.

## Figures and Tables

**Table 1:** Correlations between clinicopathological features of the cohort, c-Src, phosphorylated and dephosphorylated Y530Src expression

**Total patient cohort**	***χ*^2^ *P*-values**				
**314 Variables**	**Numbers**	**Cl28 nuc**	**Cl28 cyto**	**Cl28 memb**	**Cl28 perinuc**
Age (<50/>50 years)	78/236	0.027^*^	0.906	0.547	0.239
Tumour type (duct/lob/tub/others)	298/10/3/3	0.307	0.304	0.394	0.269
Grade (G1/G2/G3)	22/146/146	0.312	0.010^*^	0.655	0.204
Size (<20, 20–50, >50 mm)	126/169/19	0.901	0.030	0.565	0.016
Lymph node (positive/negative)	152/162	1.0	0.747	0.811	0.420
ER status (positive/negative)	209/105	0.055	0.535	0.806	0.645
PR status (positive/negative)	149/163	0.461	0.635	0.926	0.975
HER2 status (positive/negative)	51/263	0.628	0.038	0.306	0.181
c-Src nuc (positive/negative)	139/175	0.105	0.066	0.678	0.046
c-Src cyto (positive/negative)	139/175	0.740	1.0	0.811	0.629
c-Src memb (positive/negative)	135/179	0.841	**<0.001** ^*^	0.393	**0.002** ^*^
pY530Src nuc (positive/negative)	82/83	0187	0.428	0.848	0.406
pY530Src cyto (positive/negative)	73/92	0.282	**0.001** ^*^	0. 703	**<0.001** ^*^
pY530Src memb (positive/negative)	6/159	0.388	0.802	0.241	0.707
Clone28 nuc (positive/negative)	74/85	—	**<0.001**	0.037	**<0.001** ^*^
Clone28 cyto (positive/negative)	69/90	—	—	0.081	**<0.001** ^*^
Clone28 memb (positive/negative)	78/70	—	—	—	0.770

Abbreviations: c-Src=Src kinase as accessed by an antibody to c-Src; Clone28=Clone 28, Src kinase dephosphorylated at Tyrosine site 530; cyto=cytoplasm; duct=ductal carcinoma; ER=oestrogen receptor; lob=lobular carcinoma; HER2=human epidermal growth factor receptor 2; memb=membrane; nuc=nucleus; Perinuc=perinucleus; PR=progesterone receptor; pY530Src=anti-Src family negative regulatory [pY] site, Src kinase phosphorylated at Tyrosine site 530 (inactive form of Src); tub=tubular carcinoma.

The above table shows the inter-relationship between phosphorylated and dephosphorylated Y530Src expression at different cellular levels and clinicopathological characteristics of patients with breast cancer; *Histology*: others include mucinous, mucoid and micropapillary carcinoma; *Grade*: Bloom and Richardson grade. ^*^negatively correlated. Significant results are highlighted in bold. *P*-values of >0.01 were considered as non-significant due to the large number of statistical comparisons (Bonferroni phenomenon).
